# X-ray computed tomography in life sciences

**DOI:** 10.1186/s12915-020-0753-2

**Published:** 2020-02-27

**Authors:** Shelley D. Rawson, Jekaterina Maksimcuka, Philip J. Withers, Sarah H. Cartmell

**Affiliations:** The Henry Royce Institute and School of Materials, The University of Manchester, Manchester, M13 9PL UK

**Keywords:** X-ray computed tomography, Correlative microscopy, Phase contrast, Lightsheet, Time-lapse tomography, 3D imaging, 3D histology, Elemental mapping, Quantitative tomography, Water window

## Abstract

Recent developments within micro-computed tomography (μCT) imaging have combined to extend our capacity to image tissue in three (3D) and four (4D) dimensions at micron and sub-micron spatial resolutions, opening the way for virtual histology, live cell imaging, subcellular imaging and correlative microscopy. Pivotal to this has been the development of methods to extend the contrast achievable for soft tissue. Herein, we review the new capabilities within the field of life sciences imaging, and consider how future developments in this field could further benefit the life sciences community.

## Non-invasive 3D microscopy over multiple scales

**Fig. 1:**
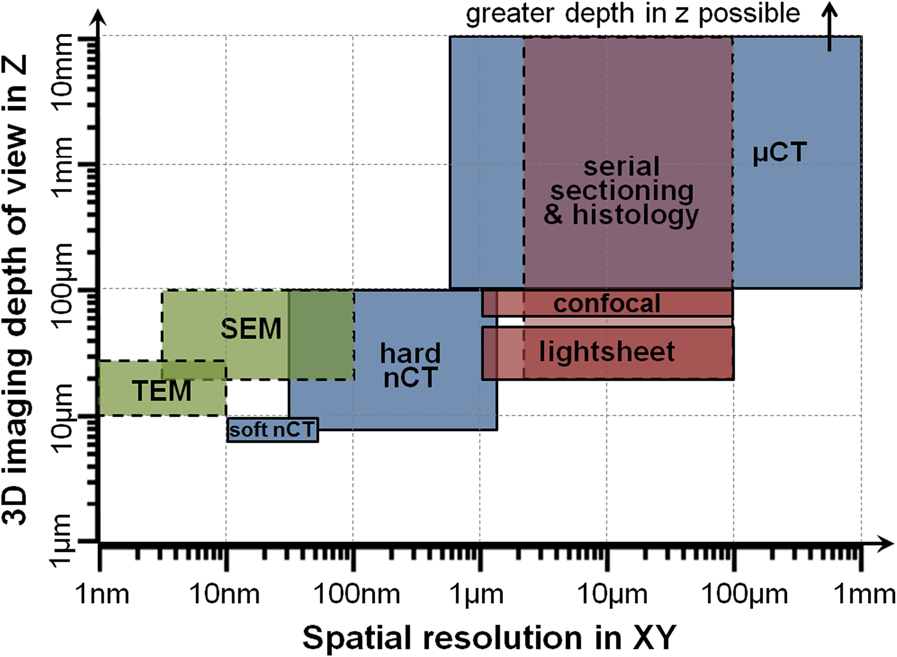
3D imaging techniques for life sciences applications, shown according to their spatial resolution (in XY) and the full depth (in Z) of the volume that can be imaged (accumulated over many serial sections for destructive methods). *Blue* = CT techniques, *green* = electron microscopy techniques, *pink* = light microscopy techniques. *Solid line* = non-invasive, *dashed line* = destructive. *TEM* serial section transmission electron microscopy, *SEM* serial section scanning electron microscopy, *Soft nCT* soft nano-computed tomography, *Hard nCT* hard nano-computed tomography, *μCT* micro-computed tomography. Data from [1–5]

**Fig. 2:**
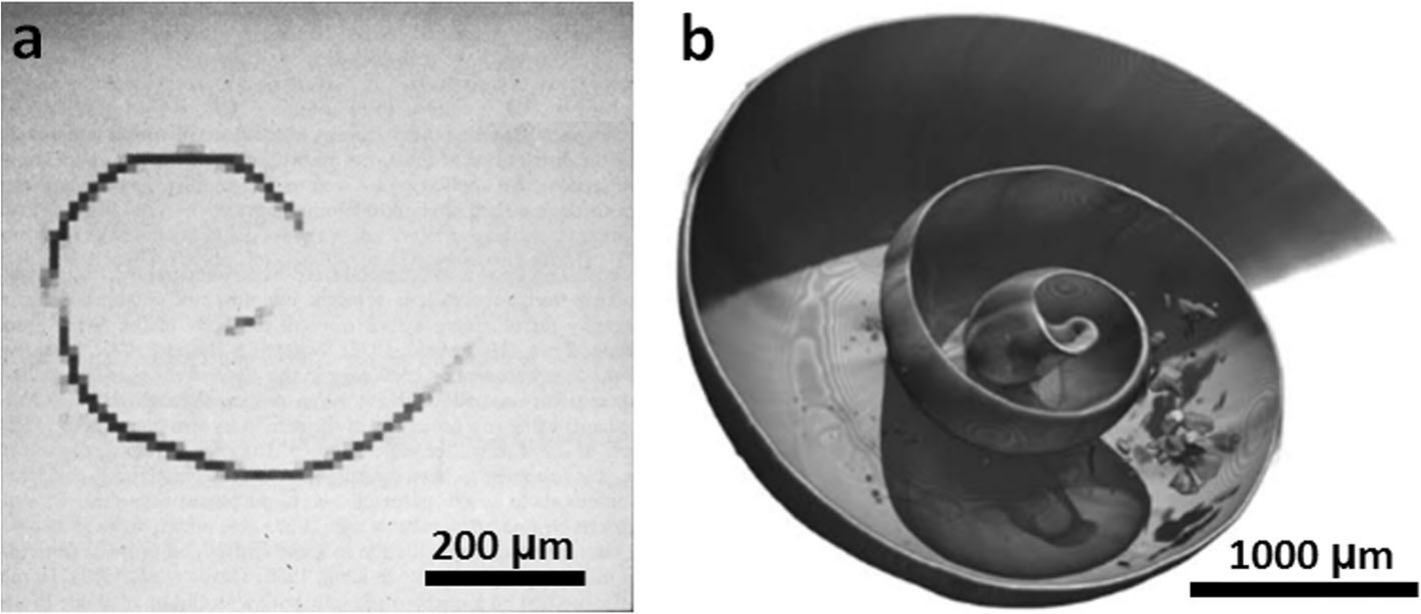
μCT imaging of *Biomphalaria glabrata* snail shell. **a** First use of μCT imaging, at a voxel (3D pixel) size of 12 μm, reproduced with permission from [6]. **b** Synchrotron μCT imaging showing the latero-frontal view of a 4-week-old snail at a voxel size of 6.2 μm with a virtual section in the median plane. Image in **b** reproduced from [7], Marxen JC, Prymark O, Beckmann F, Neues F, Epple M. Embryonic shell formation in the snail *Biomphalaria glabrata*: A comparison between scanning electron microscopy (SEM) and synchrotron radiation micro computer tomography (SRμCT). Journal of Molluscan Studies. 200,874(1);19–26, by permission of Oxford University Press

A range of 2D imaging tools, from optical microscopy to transmission electron microscopy, underpin much of what we know about structure–functionality relationships in biology, aided by a marked increase in the labels and markers available to identify certain features. To some extent, light and electron microscopy workflows have been developed to enable 3D imaging. However, they are limited by optical transparency (required for confocal and lightsheet), technically demanding sample preparation (e.g. freezing or fixing and embedding in resin followed by serial sectioning) and a limited field of view (as in transmission electron microscopy). Computed tomography (CT) exploiting the penetrating power of X-rays, on the other hand, allows non-invasive imaging of a large field of view, even for optically opaque materials, across a range of resolutions (Fig. 1), and sample preparation is comparatively straightforward. Micro-computed tomography (μCT; ~ 1 μm to > 100 μm spatial resolution) was first introduced for research applications in 1982 when Elliott et al. [6] imaged the interior of a *Biomphalaria glabrata* snail shell (Fig. 2a). Subsequent advances in μCT are evident from the corresponding image in Fig. 2b showing early stage biomineralisation of aragonite taken more recently by synchrotron μCT. These advances, alongside nano-computed tomography (nCT; down to ~ 10 nm voxel size), now allow 3D imaging from the organism level all the way down to the level of the organelles within the cell.

In essence, CT imaging involves taking many (typically over 1000) X-ray projections (digital radiographs) from different angles around a sample (typically through 360° or 180°). The X-ray projections reveal the attenuation of X-rays as they pass through the sample. The data are then computationally reconstructed, producing a greyscale virtual 3D volume of the attenuation capability of the sample. Once the 3D data set has been reconstructed, virtual slices (similar to virtual histology sections) can be extracted at any orientation and depth for viewing. Alternatively, segmentation (often on the basis of greyscale thresholding) can be used to distinguish certain constituents in 3D, allowing volumetric quantification, such as the connectivity of vascular networks [8], porosity (interconnectivity, density and pore distribution) within a biomaterial [9] or the diameter and distribution of cells within a tissue [10]. Quantification can also be undertaken by densitometric measurements, for example by comparing the attenuation of bone against a calibrant phantom to allow bone mineral density to be quantified in osteoporosis [11].

Regarding the optimal magnification and resolution for imaging a given subject, it should be noted that the spatial resolution is not equal to the voxel (3D pixel) size, but is often ~ 2–3 times larger [12]. Further, while region of interest (ROI) imaging [13] means that the sample need not be wholly within the field of view, in the majority of cases this condition is applied. This limits the effective pixel size to the sample width divided by the number of pixels across the detector, thereby limiting the resolution. Generally the attenuation of X-rays increases sharply with the atomic number of the constituents or sample size, and decreases sharply with increasing X-ray energy. Consequently, larger volume (μCT) systems tend to operate at high energy around 90–225 keV, while nCT systems generally operate below 10 keV. Soft tissue contrast improves as the X-ray energy is decreased, and so selecting the optimal energy is critical to obtaining good signal to noise ratio. CT configurations include cone beam (typical of lab μCT systems), parallel beam (typical of synchrotron X-ray systems) and helical scanning (typical of medical imaging) [14]; and magnification can be achieved either with or without lenses [15].

It should be borne in mind when imaging at high resolutions at synchrotron sources that the flux (photons/m^2^ s) can be sufficient to cause localised heating. Further, given that a certain number of photons must be detected from the imaged volume in order to reconstruct an acceptable signal to noise 3D image, the X-ray exposure (photons/m^3^) increases according to ~(1/(width of the ROI)^3^) and the time needed to acquire the image typically increases with increasing spatial resolution. The former is of particular importance when imaging live and fixed biological tissues because the increased X-ray dose associated with high resolutions can cause significant damage to soft tissues [16] and alter the mechanical properties of hard tissues such as bone [17]. Generally speaking, damage is negligible for μCT, such that live cells remain viable after imaging at micron spatial resolution [18], whereas freezing is commonplace to minimise cell structural damage when imaging at the tens of nanometres scale [19]. DNA damage of museum specimens (such as sub-fossilised bones or preserved skin) is not a concern for laboratory CT at doses below 200 Gy [20], which is unlikely to be exceeded for micron resolution imaging. Several methods have been used to minimise structural damage when imaging at high resolutions, including increasing detector efficiency, resin embedding [21], fixing and drying [22] and cryo-preservation of the samples via vitrification or high pressure freezing, which minimises cryo-damage [21, 23]. Freezing can also be followed by freeze substitution and Epon embedding (often used in electron microscopy), thereby minimising X-ray damage and preventing sample movement, but without the need to keep the sample frozen during imaging [23].

Provided X-ray damage can be avoided, the non-invasive nature of X-ray imaging presents the opportunity to track cells over time in vivo [24], observe changes in a sample over time in response to external stimuli [25, 26] or to use CT correlatively to complement other techniques. This paper reflects on recent technological advances and their application to the field of life sciences, and provides perspective on future opportunities.

## Obtaining contrast for soft tissue imaging in aqueous conditions

**Fig. 3:**
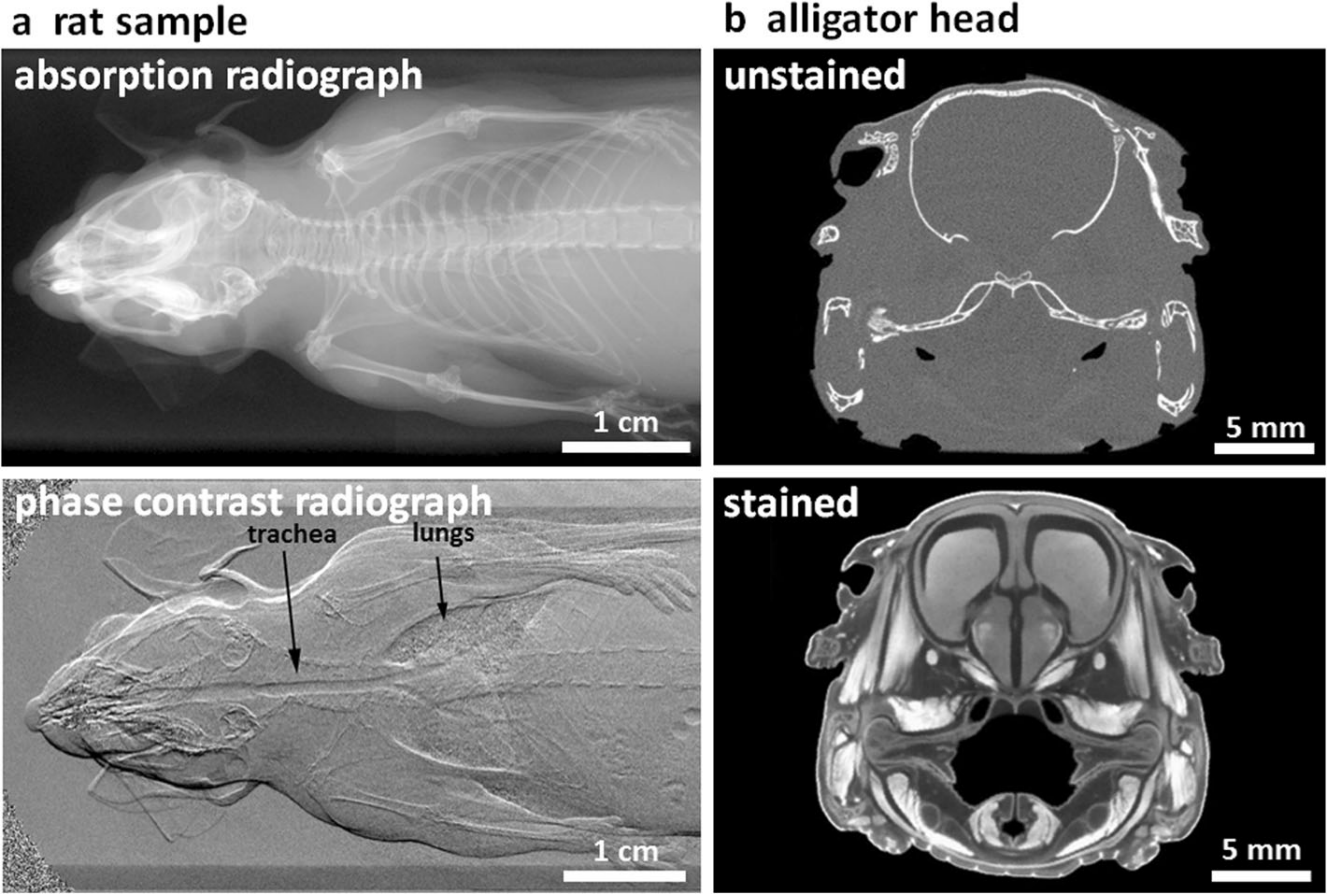
Optimising X-ray contrast. **a** A comparison of conventional attenuation (absorption) contrast and phase contrast radiographs of a rat, reproduced with permission from [27]. **b** CT section of an alligator head before and after 2 weeks of iodine staining, reproduced with permission from [28]. In **a** the conventional radiograph reveals the bone structure but not the soft tissue, whereas under grating-based phase contrast the soft tissues, including trachea and lungs, are well defined, the bones less so; in **b** only the bones are clear in the unstained sample, whereas staining reveals the soft tissues

Achieving good contrast is critically important to resolve and segment features within a sample. Life science samples present two key challenges for CT imaging. Firstly, there is typically little to no X-ray attenuation contrast between soft tissues (Fig. 3a, b). Secondly, very highly attenuating hard materials (e.g. calcified tissues or implants) positioned close to low attenuating soft tissues can result in streak artefacts which can obscure the view of the soft tissue [29].

Several techniques have emerged to obtain sufficient contrast between soft tissues, including phase contrast tomography (PCT), sample staining using heavy metals, nanoparticle labelling of cells, soft X-ray ‘water window’ imaging and dual energy CT.

In general, phase contrast (Fig. 3a) primarily enhances the visibility of the interfaces between soft tissues, such as muscle, tendon, adipose tissue and skin, along with Goods buffers and polymeric biomaterials. There are several means of achieving phase contrast [30]; however, in essence these all take advantage of the phase change generated by the refractive index as X-rays pass through the sample. For the most part, synchrotron X-ray beamlines are highly coherent, giving high contrast interference fringes, which makes PCT relatively straightforward, whereas few laboratory systems currently exhibit sufficient coherence to undertake phase contrast imaging without the use of gratings or masks [31]. PCT is being increasingly applied to the 3D imaging of soft tissues across a wide variety of applications including non-calcified musculoskeletal tissues (such as cartilage and tendon) [32, 33], the circulatory system [34] and plants [35]. PCT has proven particularly useful for fossilised specimens, where alternative means of contrast enhancement (such as staining) are not suitable [36].

Heavy metal staining can be used to improve the attenuation contrast for soft tissue CT absorption imaging. Sample staining with a heavy metal contrast agent exploits the preferential up-take of the stain by different tissues, which then attenuate more significantly in the resultant CT images [37] (Fig. 3b). While staining is well established in electron microscopy, fluorescent microscopy and histology, staining for CT imaging is in its relative infancy. To date only a few stains have been described in the literature, based mainly around those used for electron microscopy (which is also sensitive to heavy elements), including those containing iodine, tungsten, molybdenum and osmium (Table 1) [43, 45, 46]. The use of iodine has become commonplace, providing excellent contrast alongside ease of handling and cost effectiveness, and a range of staining protocols have been detailed [38]. Few studies have considered the merits of different iodine staining protocols, although iodine-ethanol solution has been found to be more effective than iodine potassium iodide at achieving greater contrast [47]. Staining duration is sample- and stain-specific; full staining is achieved in murine heart and lungs in just 3 h using an iodine or gadolinium stain [48], whereas larger specimens, such as alligator and emu heads, can require weeks of staining [28]. Mathematical models are being developed to predict the staining uptake within tissues, based on mass transfer theory [49]. However, at present a time-course study is recommended to determine the ideal staining duration of a particular tissue type with a given stain [50].

**Table 1:** Summary of heavy metal stains used to enhance soft tissue contrast in CT. Asterisks indicate the most commonly used stains

Heavy metal	Staining solution	Binds to	Staining considerations
Iodine * [38]	Iodine potassium iodide in water, ethanol or methanol. Various concentrations	Non-specific staining. Preferentially binds to lipids and glycogen. Good for muscle fibres, nerve myelin sheath, connective tissues and the eye lens	• Rapid and deep tissue penetration • Particularly suited to larger specimen (> 2 mm) • An effective stain for flower parts, allowing counting of pollen ovules [39] • Has been used to study water transport in plants [35]
Tungsten and Molybdenum* [40]	Phosphotungstic acid Phosphomolibdic acid	Proteins including fibrin and collagen. Suited to connective tissues	• Moderate tissue penetration rate and depth • Can provide more detailed visualisation compared with iodine-based stains • Phosphotungstic acid also effective in plants [41]
Osmium* [42]	Osmium tetroxide, as used in electron microscopy	Lipids including those in cell membranes, some proteins and nucleic acids	• Tissue penetration is slow and can be limited (not suited to samples requiring a penetration depth greater than 1–2 mm) • Highly toxic, requiring special safety considerations
Indium [43]	Gallocyanin-chromalum	Cell nuclei. Can show cell density and individual cells	• A histology stain • Low contrast overall
Iodine, aluminium and iron [44]	Verhoff’s Stain	Arterial walls of the vascular network	• A trichrome histology stain

Several studies have compared the relative benefits of the CT stains used to date across a range of zoological and plant specimens [43, 46]. Nevertheless, there remains great scope for further CT stain development, in terms of both the library of available stains and the characterisation of staining uptake by different tissue types. In cases where the vasculature is of interest, staining can also be achieved by the perfusion of a contrast agent through the blood vessels. This has been used to stain whole-body murine samples in just 30 min [44]. Alternatively a resin can be perfused into the blood vessels to produce a vascular cast. Upon resin polymerisation the tissue can be removed leaving only the vascular cast [51]. Whilst staining provides good tissue contrast, stains are cytotoxic and typically require prior chemical fixation to minimise tissue shrinkage [52] (Table 2). Preliminary studies should consider the shrinkage or swelling effects on the tissue or specimen of interest during selection of fixation and staining protocols. Samples may also be dried, frozen or embedded as part of sample preparation, as an alternative means of enhancing contrast or to provide stability to the samples during scanning, respectively (Table 2).

**Table 2:** Summary of sample preparation techniques

Preparation	Protocol	Considerations
Drying	Air drying, HDMS or critical point drying (for cells) and freeze drying (for tissues) [53]	• Samples become very delicate • Subsequent fixation not possible • Long or thin parts prone to movement during scanning • Compatible with electron microscopy in correlative imaging
Chemical fixation	10% neutral buffered formalin [54] 1% glutaraldehyde in acetone [55] Copenhagen mix (for plants: 70% absolute alcohol, 2% glycerol, 28% water) [41]	• Typically used before heavy metal staining to minimise sample shrinkage • Imaging may be performed in liquid or air (typically ethanol or distilled water) • Sample may move during scanning; measures to prevent movement such as packing with foam are advised • If imaging in air, measures to prevent drying are advised (e.g. placing sample in sealed container with a small reservoir of liquid to maintain humidity)
Embedding	Resin [56] or wax [34]	• Effective at preventing sample movement • Good for samples with long or thin parts which may otherwise vibrate during scanning, causing blurred imaging • Can be used with or without staining • Resin compatible with block face serial sectioning for correlative imaging
Freezing	Freezing [57] or vitrification [19]	• Use of a cryo-stage is necessary during CT imaging of frozen samples • Vitrification is typical for soft nCT as it minimises cryo-damage
Native tissue	No fixative, with staining [26]	• Used to provide contrast whilst minimising change in tissue mechanical properties (iodine in phosphate buffered saline has been used for this purpose)
	No fixative, no staining [58]	• Fix and stain unsuited to some tissues due to uptake of fluid causing swelling, e.g. intervertebral disc

**Fig. 4:**
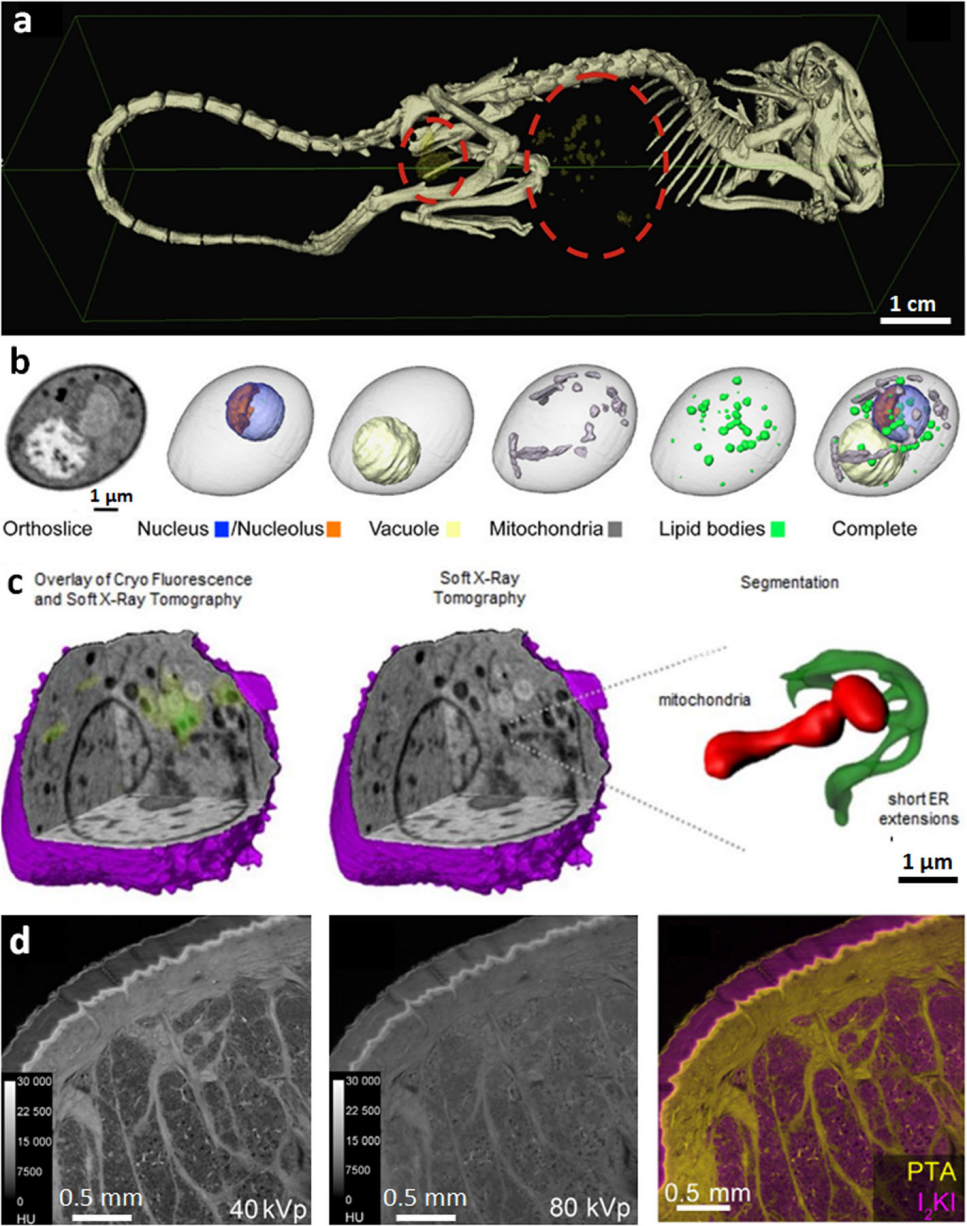
Emerging techniques for enhancing contrast in soft tissues. **a** Gold nanoparticle labelling; 3D segmented image showing clusters of gold nanoparticle labelled cells within a mouse (cells are *yellow*, circled with *red dotted line*) [59]. **b** Water window imaging showing a soft nCT section through a diploid yeast cell, the reconstructed CT volume alongside 3D representations of individual organelles and the composite image overlaying all organelles, reproduced with permission from [60]. **c** Correlative water window imaging with cryo-fluorescent microscopy; reconstructed soft X-ray tomograph of a mouse lymphoblastoid cell and overlaid cryo-fluorescence, soft X-ray tomograph alone and an expanded 3D segmented view of a mitochondrion and endoplasmic reticulum from within the cell [61]. **d** Dual energy CT; feline skin double stained with phosphotungstic acid, which preferentially stains collagen and other connective tissue (corium), and iodine potassium iodide, which stains adipose tissue (subcutaneous fat), imaged at (*left*) 40 kV and (*middle*) 80 kV, the former being more sensitive to PTA, (*right*) decomposition of the two contributions (*right*) to show adipose (*yellow*) and collagenous (*pink*) tissues, reproduced with permission from [62] Image in **a** reprinted from [59], Nanomedicine, 10(8), Astolfo A, Qie F, Kibleur A, Hao X, Menk RH, Arfelli F, et al. A simple way to track single gold-loaded alginate microcapsules using x-ray CT in small animal longitudinal studies, p.1821–8, 2014, with permission from Elsevier. Image in **c** reproduced with permission from [61], Journal of Cell Science: Elgass KD, Smith EA, LeGros MA, Larabell CA, Ryan MT. J Cell Sci, 2015;128(15):2795–804

Gold nanoparticle (~ 5 to 200 nm in diameter) labelling is an emerging technique for the labelling of live cells (Fig. 4a). It allows tracking of therapeutic cells after they have been introduced in vivo to see if they migrate to, and continue to remain at, the target site within the body [59]. The high atomic number of gold (atomic number = 79) means that nanoparticles attenuate X-rays to a much greater degree than soft tissues, providing good imaging contrast. Regarding concerns over toxicity [63, 64], many studies report that gold nanoparticles are not detrimental to the functionality of the therapeutic cell, as observed in mesenchymal stem cells, human T cells, A-431 human squamous carcinoma cells and monocytes, among others [65–67]. The ecotoxicology of nanoparticles has also been considered by observing their uptake and expulsion over time in *B. glabrata* freshwater snails [68].

For the imaging of intracellular detail, soft X-ray ‘water window’ tomography (soft nCT) is able to provide unprecedented imaging of cell organelles (Fig. 4b) at a spatial resolution better than 50 nm [69], as close to their native state as possible. Compared to hard X-rays (> 5 keV), soft X-rays (below 1 keV) with photon energies between 284 eV and 543 eV are said to be in the ‘water window’ region in which water is essentially transparent [4]. This is because X-ray photons in this energy range are absorbed an order of magnitude more readily by carbon and nitrogen in biological tissues than by oxygen in water. Using soft nCT, cells are typically vitrified, both to embed the cells in a medium and to minimise X-ray damage during imaging [19]. A variety of cellular processes have been studied to date by soft nCT [19]. Within the cell, different organelles (the nucleus, endoplasmic reticulum, mitochondrial network and plasma membrane) can be clearly identified and segmented to produce a 3D view of the organelles (Fig. 4b), determine their size [60] and identify their interactions (Fig. 4c). Until recently, soft nCT could only be undertaken on synchrotron beamlines [19]; however, laboratory-source soft nCT systems are becoming available [70].

Dual energy CT imaging can be used both to increase the contrast of soft tissue in samples containing both hard and soft tissue, as well as for identifying different stains, rather like multiple fluorescent compounds can be distinguished in optical imaging. As illustrated in Fig. 4d for imaging feline skin [62], dual energy CT involves imaging tissue first with X-rays of one energy range and then again with X-rays of a different energy range [71]. In this case phosphotungstic acid and iodine potassium iodide dual staining was used to identify collagenous and adipose tissues, respectively. Another example is dual energy imaging of a mouse toe by Handschuh et al. [62], where the percentages of hydroxyapatite, I_2_KI stain and water (background) in each voxel of the scan were identified to observe calcified tissues and soft tissues. The data sets for each material are then overlaid to produce a composite image showing the different tissue types, and segmentation can provide a 3D view of the different tissues. Lab-based spectral imaging systems incorporating photon-counting energy-resolving detectors are now available. These are able to assign photons to a small number of energy thresholds per pixel and can thus distinguish multiple stains simultaneously [72]. The logical extension of this approach is hyperspectral CT using an energy-sensitive detector to record the full X-ray energy profile at each pixel [73]. This means the absorption edges of multiple stains or key elements, e.g. Ca^2+^, can be recorded using white X-ray illumination to map the 3D distribution of certain elements. Hyperspectral imaging allows for finer energy resolution, whereby multiple elements can be identified without prior knowledge of sample composition, as demonstrated to date in materials science applications [73].

## Imaging of tissues and cells on the micro- and nano-scale

CT provides a unique view of cells as it bridges the gap between the capabilities of light and electron microscopy imaging techniques (Fig. 1). nCT can provide views of sub-cellular detail, but can also provide a large enough field of view to observe the cell in the wider context of the surrounding extracellular matrix. Indeed μCT can inform on the position, density and distribution of cells either within a tissue or on a tissue-engineered scaffold.

Nanoscale imaging can be broadly split into hard (> 5 kV) and soft (< 1 kV) X-ray nCT. As mentioned in the “Obtaining contrast for soft tissue imaging in aqueous conditions” section, the latter exploits the water window to image soft tissue. The limited penetration of soft X-rays means soft nCT is limited to a sample thickness of ~ 10 μm [74], whereas samples many tens of microns in diameter can be imaged using hard nCT at a spatial resolution down to 50 nm [15]. This provides the possibility of imaging cells within native tissues or when seeded onto biomaterial scaffolds, whereas soft nCT has typically been used to image adherent cells cultured on 2D surfaces [75], or cells in suspension [61]. For example, hard nCT has been used to observe human femur over a field of view of 9 μm containing 17 lacunae, at a voxel size of 60 nm, in which collagen fibre orientation within the bone matrix could also be identified [76]. For soft tissue imaging using hard nCT, additional techniques must be used to attain sufficient contrast, which are not necessary with soft X-rays (see the “Obtaining contrast for soft tissue imaging in aqueous conditions” section). It is possible to identify fibroblast cells and their nuclei on polymeric biomaterial tissue scaffolds using Zernike phase contrast by laboratory source nCT at 150 nm spatial resolution [22]. While individual nerve cells in tissue of the nervous system can be identified using phase contrast, osmium tetroxide staining can be used to label the myelin sheath around the axon, allowing identification of several sub-cellular structures [23]. As with CT staining more generally, staining of specific cell structures for hard nCT is in its infancy, and establishing a library of stains to label different organelles would be of great benefit.

**Fig. 5:**
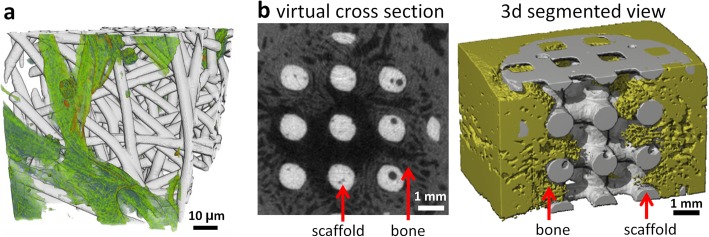
Imaging of cells and tissues on biomaterial scaffolds. **a** Segmented 3D nCT reconstruction of human fibroblast cells (*green*) on a poly (lactide-co-glycolide) (PLGA) fibre scaffold (*grey*), reproduced with permission from [22]. **b** Virtual cross-section (*left*) alongside a 3D segmented μCT reconstruction (*right*) showing bone in-growth on a hydroxyapatite scaffold after 6 weeks implantation within a critical size defect of a Yucatan minipig mandible [77] Image in **b** reprinted from [77], Biomaterials, 28(15), van Lenthe GH, Hagenmuller H, Bohner M, Hollister SJ, Meinel L, Muller R. Nondestructive micro-computed tomography for biological imaging and quantification of scaffold-bone interaction in vivo, p.2479–90, 2007, with permission from Elsevier

μCT is proving particularly useful for the imaging of biomaterial tissue scaffolds intended to replace and regenerate tissues and organs of the body. These materials are not generally optically transparent or easily sectioned, making their 3D analysis challenging by light and electron microscopy. Scaffolds may be ceramic, metallic, polymeric or hydrogel, and extracellular matrix (ECM) produced by the cells may be made of calcified or soft tissues. μCT imaging is well suited to analysing the formation of mineralised tissue on polymeric and ceramic scaffolds as mineralised tissue has a high X-ray absorption and so can be easily distinguished using conventional X-ray attenuation imaging [77] (Fig. 5b). Quantitative 3D data can be obtained, including scaffold porosity, interconnectivity, volume and surface area, along with calcified tissue volume, distribution and density [18]. μCT imaging of soft tissue production on a polymeric scaffold or hydrogel poses a greater challenge compared with mineralised tissues since polymers and soft tissue attenuate X-rays similarly, resulting in poor X-ray contrast. This has been addressed by the use of either heavy metal contrast agents [78] or PCT imaging (Fig. 5a) [22]. Quantitative analysis has been undertaken of the depth and distribution of cellular infiltration into the scaffold [78]. Imaging of non-mineralised ECM production on metallic biomaterials presents different challenges. While high energy X-rays are typically used to image metals, the low X-ray attenuation associated with soft tissues at high energies mean that they cannot easily be discerned. For these applications, protocols have been developed to increase the X-ray opacity of the developing non-mineralised ECM, allowing visualisation of the ECM formation using high-energy X-rays [79].

## Correlative imaging

Correlative light and electron microscopy exploits light microscopy to image fluorescent markers and identify particular molecules as well as proteins (e.g. [80]), whilst electron microscopy provides the ultrastructural context. Similarly, correlative CT exploits multiple techniques or imaging modalities to obtain different types of information from a given ROI or volume of interest [81]. Data can also be correlated across images acquired at different times (temporal correlation), for example to observe mineral formation over time in 3D scaffolds [18], or across multiple length scales using multi-scale CT, sometimes termed zoom tomography, as used to identify the location of macrophages within mouse lung [16].

Conventional histological analysis has been combined with μCT as a means of both validating CT and also to verify the different tissue types within a given sample [82]. During the correlative study of cartilage, PCT-enhanced μCT surpassed histological analysis: comparable spatial resolution was achieved across both techniques but μCT provided 3D data [83]. Another application for correlative μCT with light microscopy is to assess both cell viability and 3D cellular infiltration in biomaterial applications. Soluble assays are used to determine cell viability, followed by μCT, which provides complementary data on cell infiltration and distribution within the scaffold in 3D, often unattainable by other means, thus allowing a richer understanding of the samples [84]. Similarly, 3D nCT has been used for the imaging of cells alongside optical microscopy of fluorescent labels to identify organelles within the cell [85].

μCT has also been proposed as a means of experimental steering ahead of more detailed and time-consuming serial block face SEM [56]. Serial block face SEM involves the automated sequence of material removal (via ultramicrotome or focussed ion beam milling) of a stained and embedded sample followed by SEM imaging of the cut surface of the block to construct a 3D volume from the imaged slices, e.g. of tendons [86]. 3D volumes of 50-μm dimensions are commonly imaged by serial section focussed ion beam milling and SEM, while volumes of 500 μm are common by ultramicrotomy and SEM. CT experimental steering can be used to enable high-throughput pre-screening of multiple samples, to identify samples containing specific rare features, to ensure adequate sample preparation prior to serial block face SEM studies, to steer 3D serial sectioning workflows, to locate specific features for detailed site-specific investigation, to provide an overview of the sample from which higher-resolution electron microscopy images are acquired or to assess the extent of sample preparation artefacts [56].

**Fig. 6:**
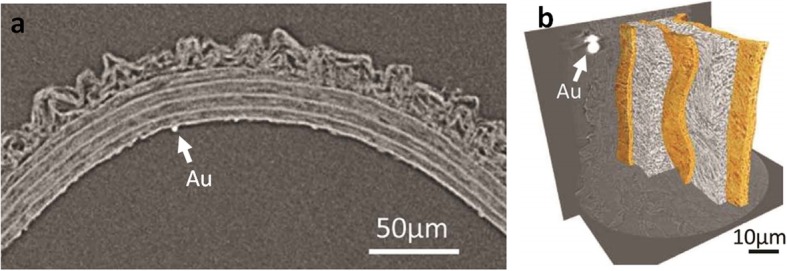
Gold fiducial marker (Au) in a rat aorta, allowing co-registry of multi-scale CT imaging, reproduced with permission from [34]. **a** Virtual cross-section through μCT data at a spatial resolution of 0.7 μm. **b** Segmented reconstruction of nCT data at 150 nm spatial resolution

To ensure appropriate registry when undertaking multiscale correlative imaging, fiducial markers (e.g. gold or tungsten carbide particles) are often used when transferring samples between instruments to locate the same ROI and to align the resulting data sets. A fiducial marker can take any form, either an intentional notch or scratch in the sample or an embedded small item or particle, which can be identified from the CT scan. For example, Zehbe et al. [83] used a screw as a marker to ensure imaging of the exact same cell with both tomography and subsequent light microscopy of stained histological sections. Similarly, Walton et al. [34] used gold beads (1–3 μm diameter) as fiducial markers to locate the same region within the rat aorta wall (Fig. 6).

**Fig. 7:**
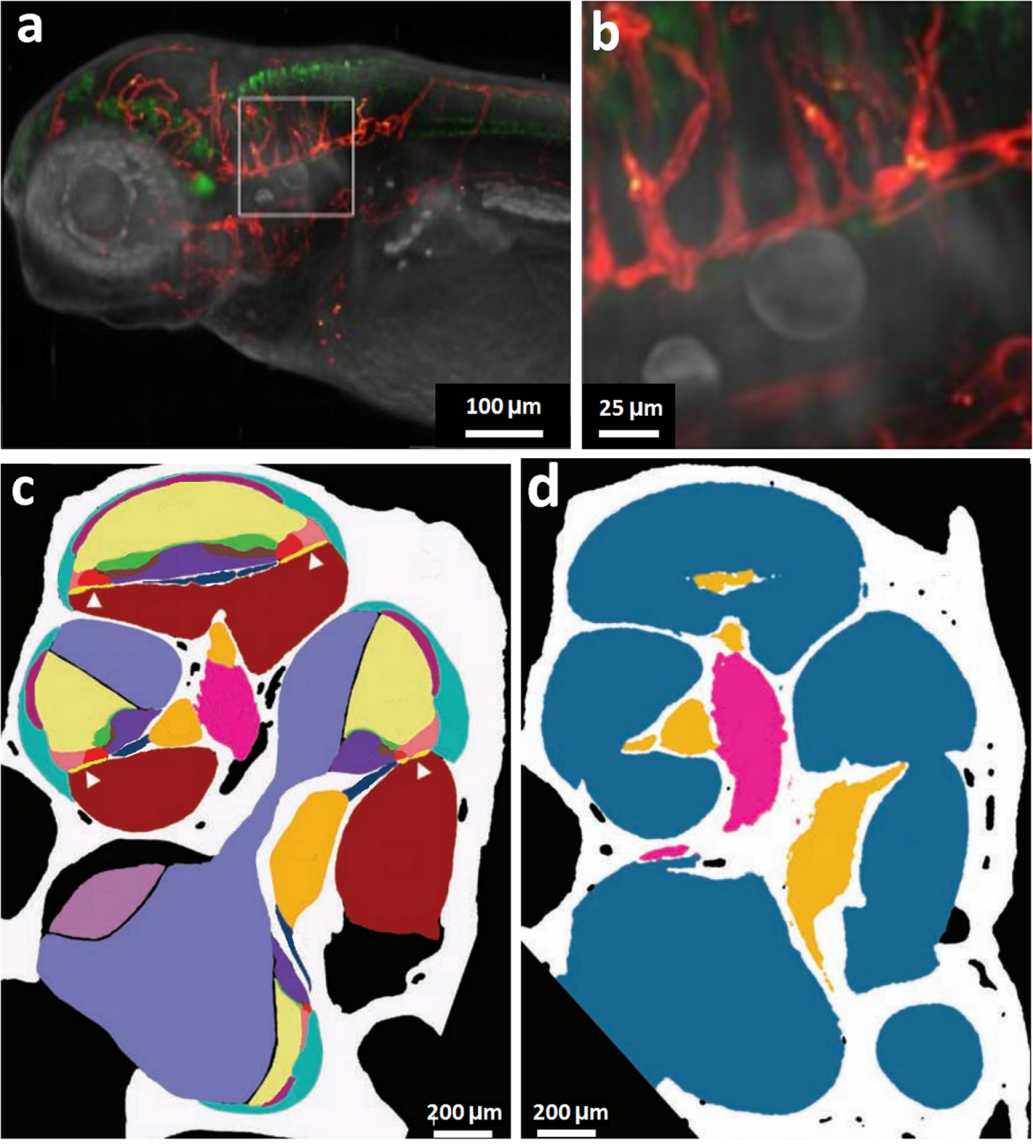
LSFM with complementary μCT and visible light tomography. **a, b** Zebrafish (lateral view) imaged using correlative LSF and visible light tomography, showing **a** head of the zebrafish and **b** larger view to show detail. *Red* = vasculature, *green* = nervous system [89]. **c, d** Cross-section through the segmented reconstruction of the midmodiolar section of the mouse right ear, imaged using **c** LSFM and **d** μCT. From the LSFM data, 15 tissue types can be identified: bone (*white*), spiral ligament (*turquoise*), saccule (*pale purple*), stria vascularis (*dark purple*), tectorial membrane (*green*), scala media (*cream*), basilar membrane (*yellow* with *white arrowhead*), Rosenthal’s canal (*orange*), Claudius cells (*pale pink*), modiolus (*bright pink*), organ of Corti (*bright red*), scala tympani (*dark red*), scala vestibuli (*pale blue*), spiral limbus (*mid-blue*), osseous spiral lamina (*dark blue*). From the μCT data, four tissue types can be identified: bone (*white*), cochlea scalae and vestibular labyrinth (*blue*), Rosenthal’s canal (*orange*) and modiolus (*pink*) [90] Images in **a** and **b** reproduced with permission from [89], Development: Bassi A, Schmid B, Huisken J, Development, 2015, 142(5):1016–20. Images in **c** and **d** reproduced with permission from [90]

Whilst in its infancy, correlative lightsheet fluorescent microscopy (LSFM) and CT imaging can enable the imaging of small and large features, respectively [87]. During LSFM imaging, the sample is illuminated with a laser, which is focused to a sheet of light only a few microns in thickness, and the detection axis is perpendicular to illumination. In-focus light is imaged simultaneously rather than pixel by pixel as in conventional confocal microscopy, and the narrow plane of focus of the laser also reduces phototoxicity and photobleaching [88]. Lightsheet has been applied to studying nervous system development in the transparent zebrafish, which has been correlated with visible light tomography to provide anatomical context (Fig. 7a, b) [89]. A limited number of studies have sought to compare LSFM with μCT, observing the murine cochlea [90] or carotid artery and micro-vascular networks [87]. Buytaert et al. [90] correlated LSFM data to μCT in the study of the mouse cochlea and obtained high-resolution images of anatomical, morphological and histological organisation of soft tissue from LSFM, the results of which were comparable to those obtainable from histological analysis (Fig. 7c, d). The μCT data were used correlatively alongside LSFM to provide more accurate dimensional information, which is especially important to combat the effects of shrinkage from LSFM sample staining [90]. A limitation of LSFM is the requirement for optically transparent samples. Opaque tissue samples must be cleared to increase optical penetration depth, but the clearing process can lead to a significant amount of tissue shrinkage [91]. As an alternative to clearing, advances in equipment are now available that can pivot the lightsheet to provide improved imaging for more opaque samples [92].

Given that the form and function of biological tissue is determined from the molecular up to the whole-organism scale, multi-scale CT can be particularly useful. The whole sample can be mapped at the highest magnification, but it is more often experimentally and data efficient to follow a targeted trajectory tracking specific ROIs [93]. In some cases it is possible to traverse the scales non-invasively using ROI scanning [13] to investigate ROIs at increasing magnifications. In other cases it is necessary to remove a ROI for investigation by CT or electron microscopy at successively higher resolutions. Xenon plasma focussed ion beam milling has proven an effective technique for excising suitable regions of interest [3] as it provides accurate material removal at a rapid rate when compared with conventional gallium focussed ion beam milling. One challenging aspect of correlative imaging is identifying the same area of interest for subsequent scans when transferring a sample between equipment (when moving from μCT to nCT for example). Fiducial markers are typically used for this purpose (e.g. surface markings [94] and metallic particles). Walton et al. applied multiscale CT imaging to the study of rat arterial walls using micro- and then nano-PCT of unstained, wax-embedded samples [34] (Fig. 6). From the nCT (150 nm spatial resolution), individual medial lamellae could be identified and segmented, whilst μCT provided wider context to their arrangement within the whole artery [34]. Similar multiscale work has been performed in the study of human tooth dentine [16, 94].

**Fig. 8:**
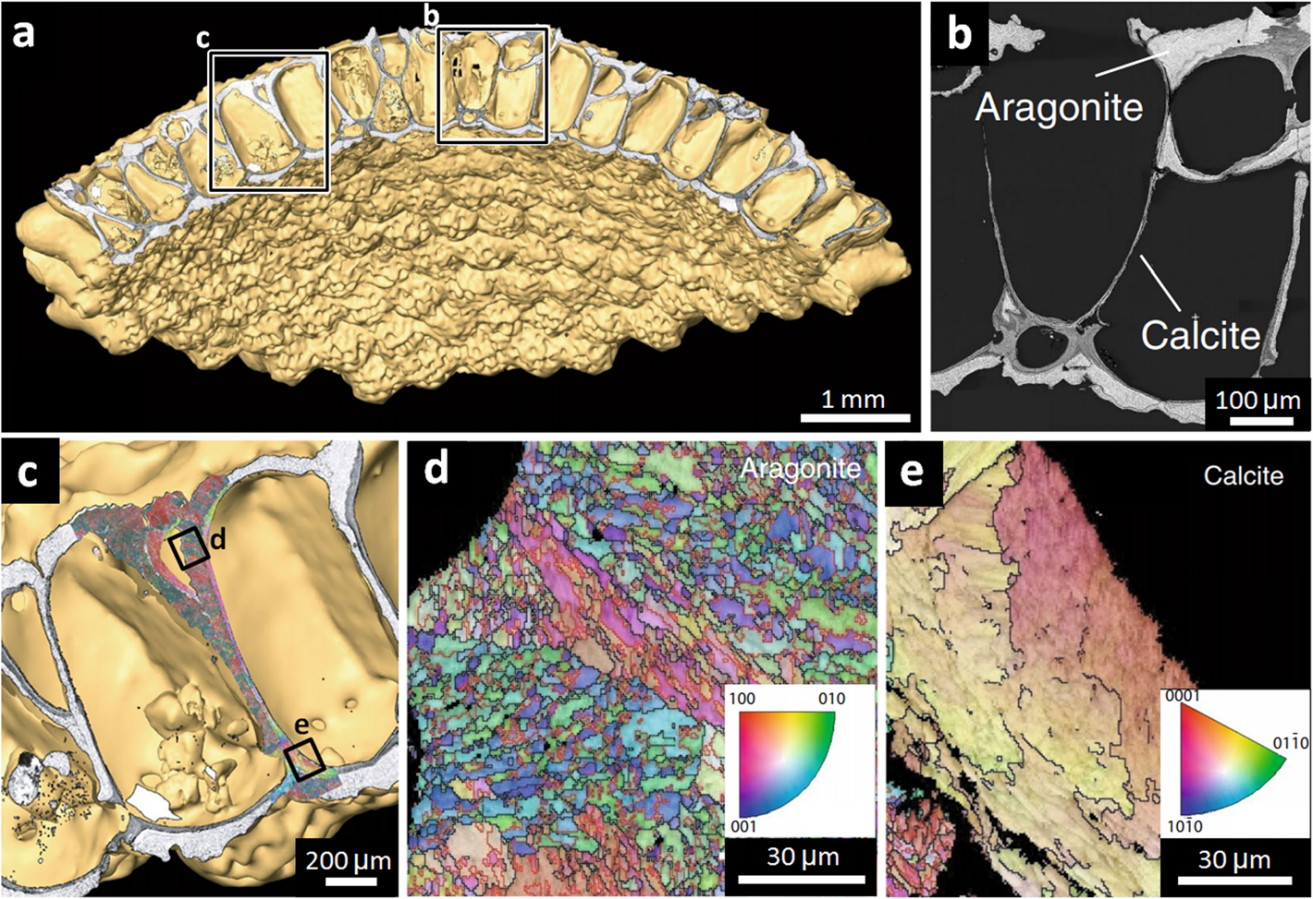
Correlative microscopy of the anoteropora latirostris (saltwater invertebrate) colony, reproduced with permission from [95]. **a** CT of the colony. **b** Backscattered electron imaging showing aragonite and calcite regions. **c** Electron backscatter diffraction overlaid onto CT volume data. **d**, **e** High-resolution electron backscatter diffraction data, showing crystallographic grain structure in the **d** aragonite and **e** calcite regions

When considering biominerals, studies have used backscattered electron imaging and electron backscatter diffraction to determine areas of different mineral phases and grain crystallographic orientation, respectively, as exemplified in the study of aragonite and calcite crystal forms of calcium carbonate in *Anoteropora latirostris* (saltwater invertebrates which live in interconnected colonies) [95]. Correlating electron microscopy with subsequent μCT is particularly useful in providing wider context for the crystallographic data, demonstrating how it relates to the wider architecture of the *A. latirostris* colony, allowing inference towards biomineral deposition during colonial development (which begins at the colony centre) (Fig. 8) [95]. Further to this, X-ray diffraction can inform on grain structure and orientation in 3D. Correlative X-ray diffraction and μCT has been applied to fossilised bone samples to determine hydroxyapatite orientation and infer muscle attachment sites, previously only achievable destructively using polarised light microscopy after sample sectioning [96]. Multi-modal data correlation is challenging, particularly when spanning several orders of magnitude and when involving 3D data sets; however, developments in software are approaching automated correlation, making data management more practical [93]. Correlation software has been used in the study of the acorn barnacle *Semibalanus balanoides*, using light microscopy, μCT, scanning electron microscopy, scanning electron microscopy and electron backscatter diffraction correlatively, spanning several orders of magnitude [97].

## Tracking microstructural changes within a sample over time

Since CT is non-invasive, sequences of images can be acquired to observe changes over time, termed time-lapse μCT. For example, temporal studies are improving our understanding of biomaterial degradation in a fluid-flow environment [98], water transport in plants [35] and plant root growth [99]. These techniques have also been applied to tissues that require mechanical stress for homeostasis, such as musculoskeletal tissues [25], or respond to internal pressure, such as arteries [34]. Mechanical loading studies are also of interest for investigating damage to tissues [100], or the damage tolerance of an implanted device [26], for instance.

Live cell imaging over time is of particular relevance for the tracking of therapeutic cells in vivo and for the analysis of tissue-engineered (TE) scaffolds. For biomaterials research, longitudinal time-lapse studies are possible where either conventional X-ray attenuation imaging or PCT imaging provides sufficient contrast such that no toxic stains are necessary and the dose is not sufficient to affect the viability of the cells. In the case of high attenuating calcified tissue on polymer or ceramic scaffolds, X-ray attenuation imaging has been used to identify scaffold degradation and tissue volume, infiltration and density over time during in vitro culture [18]. For soft tissues on polymeric and hydrogel scaffolds, which exhibits poor contrast during X-ray attenuation imaging, PCT has allowed quantitative analysis of biomaterial degradation and integration into host tissue over time in a subcutaneous murine in vivo study [101]. Due to repeated X-ray exposure, retaining cell function is of concern for time lapse μCT imaging of cellularised scaffolds both in vitro and in vivo. One study observing mineralised matrix formation by rat stromal and calavarial cells reported no reduction in mineralised matrix formation resulting from weekly μCT scans at 16 μm isotropic voxel size [18]. Conversely, at higher spatial resolutions (50 nm), studies have found structural damage to samples [16], which can be minimised by freezing; however, this introduces added concerns over freeze-thaw damage in potential longitudinal studies. There is currently no recommended X-ray limit for longitudinal studies; inroads have been made towards identifying X-ray settings that impose minimal dosages whilst producing adequate scans for TE and in vivo applications, specifically for a cartilage TE implant in a porcine knee joint [102]. Further in vivo studies for wider TE products would be beneficial, as would establishing guidelines on the maximum X-ray dosage that does not alter in vitro cellular function.

**Fig. 9:**
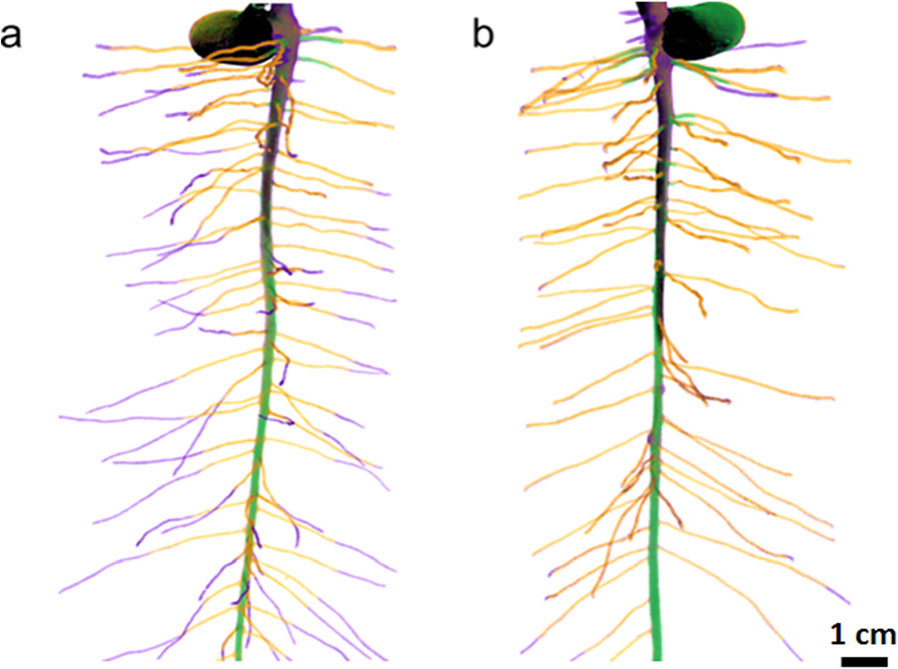
Time-lapse imaging of a fava bean root showing impaired growth with increased imaging rate, reproduced with permission from [99]. **a** Imaged every 2 days. **b** Imaged every 4 days. Colour represents number of days after planting: *black* = 4, *green* = 8, *orange* = 12 and *purple* = 16

Time-lapse μCT has also proven useful in the study of developing root systems, resulting in greater understanding of the root–soil interface in different soil types, and unexpectedly demonstrating reduced root–soil contact and increased soil porosity over time [103]. As with longitudinal studies of live cells, X-ray exposure is a concern. For live plants, a recommended cumulative dose limit of 33 Gy has been proposed [104]; however, some plant species are particularly sensitive to X-rays. The date and plum exhibit impaired germination at 0.05 Gg [104] and the fava bean shows reduced root and leaf growth in longitudinal studies reaching a cumulative dose of 8 Gy (Fig. 9) [99]. Caution is therefore advised, and unexposed controls are recommended.

## CT imaging considerations

Several practical aspects with regards to CT imaging must be considered, including acquisition time, cost, equipment access, image processing and data management.

In general, as spatial resolution is increased the required scan time also increases (see the “Obtaining contrast for soft tissue imaging in aqueous conditions” section). Whereas laboratory-based X-ray μCT may take a few hours, nCT can be expected to require at least double this amount of time [105]. However, acquisition time limitations can largely be overcome by exploiting the high brightness of synchrotron X-ray sources. Depending on the required resolution, as many as ten scans per second can be acquired [12]. High frame rate CT imaging generates very large amounts of data very quickly, which can be challenging to manage, and which takes considerable time and effort to analyse. Given that there are relatively few large scale synchrotron facilities globally, access is limited and often restricted to a few days at a time, prohibiting certain longitudinal studies.

Segmentation of 3D μCT and nCT data sets can be time-consuming, especially if manual intervention is required to delineate different regions or phases within each slice in turn. In cases where time-lapse data are acquired, quantification is necessarily reliant on automated segmentation and quantification procedures because of the sheer volume of data to be analysed in 4D. Looking forwards, segmentation of CT datasets is set to benefit from the emergence of machine learning techniques, which are currently in development within the medical field for disease recognition and to approach personalised medicine [106] as well as in other fields of computer vision analysis [93].

## Conclusions and future perspectives

The capability of laboratory and synchrotron CT imaging is increasing apace in terms of acquisition rate, spatial resolution and sensitivity [12]. At present, laboratory source nCT and high resolution μCT can take hours or days to acquire a scan while those at tens of microns can take seconds or minutes. The brilliance of synchrotron sources mean acquisition rates can be some 1000 times faster, meaning that at present fast acquisition is best undertaken at synchrotrons, whereas longer or dose-sensitive samples may be better suited for laboratory μCT. Increases in acquisition rates at laboratory sources would be particularly beneficial for imaging of fresh, un-fixed specimens, and to allow practical imaging of replicates of samples and during longitudinal studies where multiple scans are necessary. This can be achieved by reducing the number of projections taken through the 360° rotation of the sample, or by reducing the projection time, which in turn reduces X-ray counts. Iterative reconstruction algorithms are now available to allow reliable reconstruction of tomography data obtained in shorter times, with no significant detriment to imaging quality [107].

The efforts to improve speed, spatial resolution and sensitivity may also provide the possibility of imaging at the same resolution as currently available, but at lower X-ray dose. This is of specific concern for biological specimens during longitudinal studies where repeated exposure is necessary and of increasing relevance at greater spatial resolution. Whilst reducing X-ray exposure would undoubtedly be beneficial, the literature on the effects of X-rays (of different energies) on cells during μCT and nCT imaging is sparse with no specific guidance on exposure limits for research. Establishing the X-ray dosage levels that cause either a decline in function or cell death, across a variety of cell lines, would be beneficial fundamental research to provide confidence in longitudinal studies where cells are repeatedly imaged.

Correlative imaging, either between multiple machines or within single instruments, is becoming more popular. The key challenge for correlative tomography is the ability to co-register and combine data from multiple modalities. Software is currently available that enables alignment of predominantly 2D data sets for correlative light and electron microscopy, and has some 3D capability to correlate between focussed ion beam SEM, CT and light microscopy; however, software allowing improved automated correlation of 3D data sets is under development [108].

Improving the efficiency and automation of the segmentation stage of data analysis would be particularly beneficial from a practical perspective, allowing a greater number of sample replicates to be analysed in a practical amount of time. Automated segmentation processes are currently available, but these are not effective when there is high noise or poor contrast in the data. Efforts to improve imaging quality (improved sensitivity, improved contrast and reduced noise) may enable automated segmentation with greater ease. In addition, development is ongoing into improving the automated segmentation capabilities of software through methods such as machine learning [109]. The application of CT to the life sciences is increasing, complementing traditional light and electron microscopy, and is likely to increase further with further advances in capability and analysis procedures.

## Data Availability

Not applicable.
